# Damage-free vibrational spectroscopy of biological materials in the electron microscope

**DOI:** 10.1038/ncomms10945

**Published:** 2016-03-10

**Authors:** Peter Rez, Toshihiro Aoki, Katia March, Dvir Gur, Ondrej L. Krivanek, Niklas Dellby, Tracy C. Lovejoy, Sharon G. Wolf, Hagai Cohen

**Affiliations:** 1 Department of Physics, Arizona State University, Tempe, Arizona 85287, USA; 2 LeRoy Eyring Center for Solid State Science, Arizona State University, Tempe, Arizona 85287, USA; 3 Laboratoire de Physique des Solides, Université Paris-Sud, CNRS, UMR8502, Orsay 91405, France; 4 Department of Structural Biology, Weizmann Institute of Science, Rehovot 76100, Israel; 5 Nion Co., 11511 NE 118th St., Kirkland, Washington 98034, USA; 6 Department of Chemical Research Support, Weizmann Institute of Science, Rehovot 76100, Israel

## Abstract

Vibrational spectroscopy in the electron microscope would be transformative in the study of biological samples, provided that radiation damage could be prevented. However, electron beams typically create high-energy excitations that severely accelerate sample degradation. Here this major difficulty is overcome using an ‘aloof' electron beam, positioned tens of nanometres away from the sample: high-energy excitations are suppressed, while vibrational modes of energies <1 eV can be ‘safely' investigated. To demonstrate the potential of aloof spectroscopy, we record electron energy loss spectra from biogenic guanine crystals in their native state, resolving their characteristic C–H, N–H and C=O vibrational signatures with no observable radiation damage. The technique opens up the possibility of non-damaging compositional analyses of organic functional groups, including non-crystalline biological materials, at a spatial resolution of ∼10 nm, simultaneously combined with imaging in the electron microscope.

## Abstract (web summary)

Use of electron microscopy to determine morphology, or find where functionally significant biomolecules are located with high spatial resolution is of great interest. Here, Rez, Cohen *et al*. use aloof electron beam vibrational spectroscopy to probe different bonds in biological samples with no significant radiation damage.

## Graphical abstract

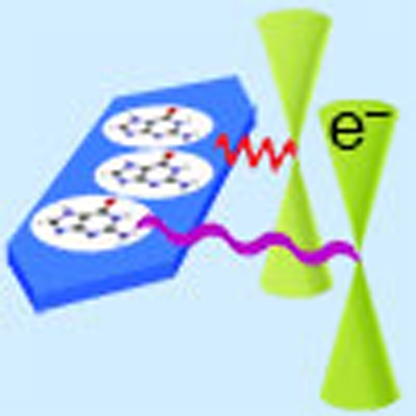

A central paradigm in biology is the inseparable connection between structure and function of biogenic molecules. Imaging of vitrified biological cells, tissues and macromolecules by transmission electron microscopy (TEM) has progressed rapidly in extracting complex structural information^1^,^2^,^3^. These techniques provide structural information, but do not probe the sample properties. They are also insensitive to the presence of hydrogen, the most common element in organic molecules, and to how hydrogen is bonded to other atoms. Although it is possible to acquire infrared spectra at a spatial resolution of better than 100 nm using tip-enhanced infrared absorption in an atomic force microscopy^4^,^5^, the technique is limited to films that are flat over an extended region. Selecting the amide I absorption peak, Berweger *et al*.^6^ showed that it was possible to map the distribution of bacteriorhodopsin in phospholipid bilayer. If spectroscopy carried out in the electron microscope could be extended to the visible and infrared region, then it should be possible to use electron microscopy to not only determine overall morphology but also to find where functionally significant biomolecules, such as chromophores and proteins, are located within the overall structure at high spatial resolution.

The damage an energetic electron beam causes to the biological sample is another key limitation of present-day electron microscope imaging and analysis. Avoiding the damage requires that the electron fluence be kept at a very low level, typically of the order of <10 e Å^−2^ (refs 7, 8), and this severely limits the spatial resolution at which biological samples can be imaged and analysed^9^. Methods for minimizing the radiation damage include averaging the information over many unit cells of a periodic sample, averaging over many identical structural units in a non-periodic sample^10^ examining cryogenically preserved specimens at liquid nitrogen temperatures (cryo-TEM)^11^ and using direct electron detectors to fractionate dose among many image frames^3^,^12^,^13^.

Recent developments have demonstrated electron microscope resolution of infrared features, by electron energy loss spectroscopy (EELS) carried out with an energy resolution of ∼10 meV (ref. 14). This has made it possible to record spectra showing vibrational features of hydrogen-containing inorganic materials such as metal hydrides, but the approach has not been applied to biological samples. Aloof beam spectroscopy^15^,^16^ was proposed by Cohen *et al*.^17^ as a near-field spectroscopy probe and was used by Krivanek *et al*.^14^ to acquire vibrational spectra from titanium hydride. The theoretical advantage of using aloof beams to avoid radiation damage has recently been explored by Egerton^18^. The key advantage of aloof spectroscopy is that for a tightly focused beam passing a distance *d* outside the sample, damage-causing high-energy interactions are suppressed relative to information-providing low-energy interactions. In other words, an ability to accurately control the beam–sample distance has an important advantage: one can choose the effective range of energy transferred to the sample, similar to what can be achieved with photon techniques by adjusting the beam energy range. Thus, it is not just the beam intensity, or scattering cross-section that is varied, but the fundamental characteristics of beam–sample interaction. Specifically, by selecting *d*-values that do not allow energy transfers above the infrared regime, one can perform measurements that are, in principle, similar to probing with infrared irradiation, hence with minimal damage.

Here we show that aloof electron beam vibrational spectroscopy can be carried out at an energy resolution sufficient to probe different types of bonds in biological samples, including those of hydrogen, carbon, nitrogen and oxygen, with no significant radiation damage.

## Results

### Vibrational spectroscopy

**Figure 1:**
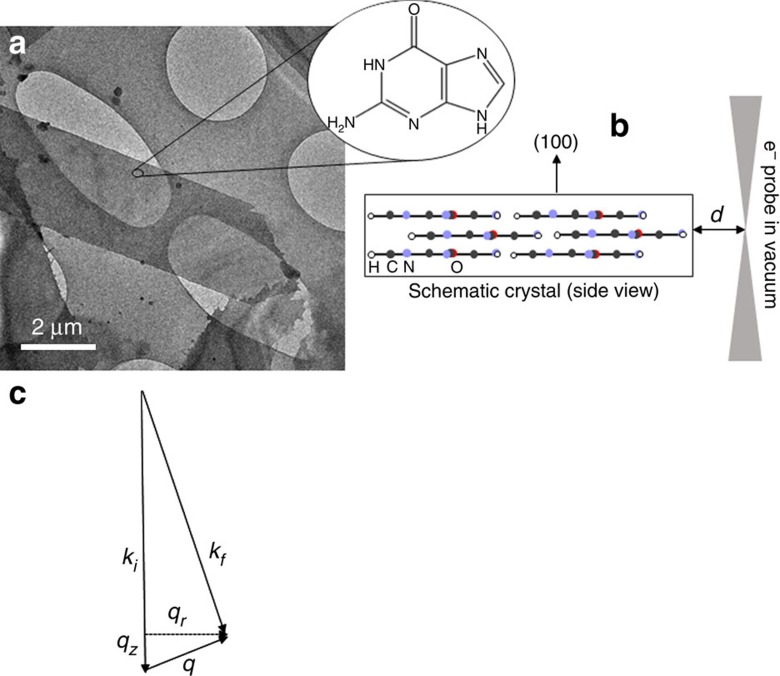
Position of the electron beam with respect to the guanine crystal. (**a**) Low-magnification ‘top-view' image of guanine crystals laying on holey carbon film. The long edges of the crystal are in the (010) direction using the unit cell of Hirsch *et al*.^37^ (**a**, inset) one schematic guanine molecule—the molecules are arranged in layers parallel to specimen surface to form the crystal as shown schematically from the side in **b**. Also shown in **b** is the position of the electron probe a distance *d* outside the crystal in the vacuum. (**c**) Schematic diagram showing the scattering wavevector for the electrons (side view). The energy loss is small compared with the energy of the incident electrons and the scattering angles are also small. The scattering wavevector can be decomposed into a component *q*_*z*_ along the incident beam direction related to the energy loss (equation 1) and *q*_*r*_ perpendicular to the incident beam direction. Under our experimental conditions, *q*_*r*_ is much larger than *q*_*z*_.

To demonstrate control over beam–sample interaction, we apply the aloof beam EELS configuration (Fig. 1b) for the detection of electronic and vibrational peaks in guanine, one of the DNA bases. Anhydrous guanine in its crystalline form has a strong anisotropic dielectric response that is used in nature by organisms such as fish^19^,^20^, arthropods^21^ and reptiles^22^,^23^ for manipulating light. Crystals extracted from the scales of the Japanese Koi fish (*Cyprinus carpio*) were purified and placed on Cu holey carbon-coated TEM grid (see Methods for details). They are in the form of rhombohedral plates ∼10-μm long and 30-nm thick. A typical guanine crystal is shown in the TEM image (Fig. 1a). It is lying on a holey carbon film, such that the e-beam of a Nion UltraSTEM can be directed through vacuum at a distance *d* from the side of the crystal that can be accurately controlled in a range of 10–250 nm, as shown schematically in Fig. 1b. The scattering wavevectors perpendicular to the beam direction *q*_*r*_ in the plane of the specimen are much greater than *q*_*z*_ under our experimental conditions (Fig. 1c). Also, under our experimental conditions, dipole scattering dominates^24^. The planar guanine molecules (inset in Fig. 1a) with 1 C=O bond, 1 NH_2_ group, 1 CH bond, 2 NH bonds and 7 C–N bonds all in the same plane are stacked parallel to the surface of the crystal (Fig. 1b). This means that we selectively excite vibrations in the plane of the guanine molecule.

**Figure 2:**
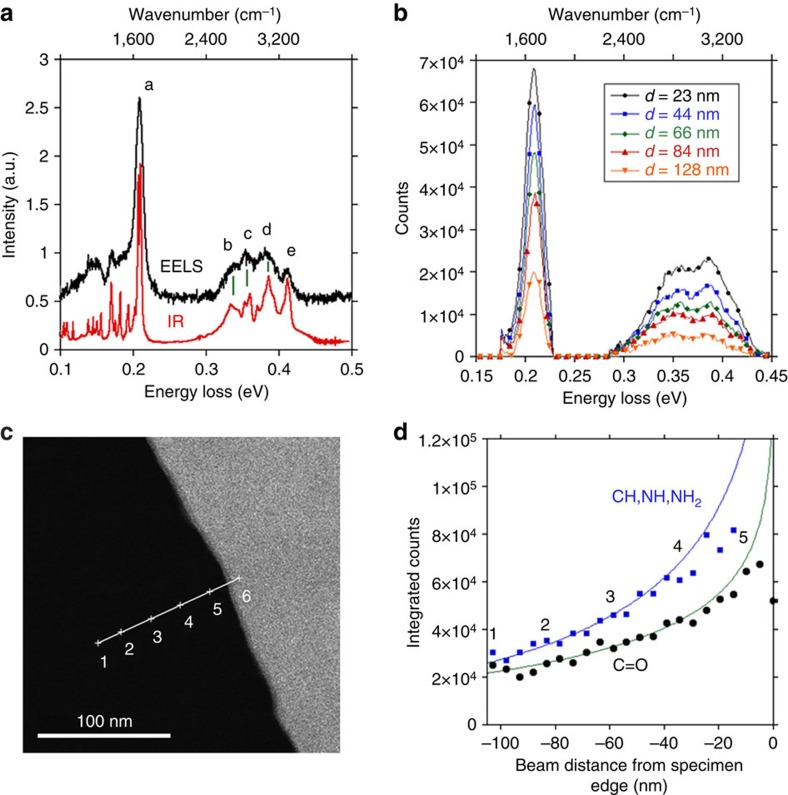
Variation of infrared region spectra with electron beam position. (**a**) An EEL spectrum at the infrared region, collected when the electron probe is 30 nm from the edge of the crystal, compared with an *ex situ* FTIR spectrum. Peaks corresponding to C=O, NH, CH , NH_2_ symmetric and NH_2_ antisymmetric stretches can be seen in the EEL spectrum, matching corresponding features in the FTIR (Table 1). (**b**) A set of spectra showing how the peaks in the infrared region increase in height as the probe is moved closer to the specimen. (**c**) Dark-field image showing guanine crystal–vacuum interface and line along which spectra were taken. (**d**) Variation of total intensity of the C=O peak (circles) and the combination of CH, and NH and NH_2_ peaks (squares, peaks b–e from (**a**)) from the EELS signal as the probe approaches the sample compared with the theoretical variation derived from classical dielectric theory given as equation (2) (solid lines). The acquisition time was 1.6 s a point.

Figure 2a presents an EEL spectrum in the infrared range, as recorded at a distance *d*=30 nm from the crystal edge with beam energy of 60 keV. Close agreement with the infrared absorption spectrum, Fig. 2a, of anhydrous guanine crystals (acquired *ex situ*) is found, including the detailed peak positions given in Table 1. The most prominent feature is the peak from the C=O stretch at 209 meV. All the peaks that can be attributed to stretching of hydrogen covalently bonded to nitrogen or carbon have been identified in the spectrum as shown in Table 1. Thus, this spectrum demonstrates not only the detection of hydrogen but also the capability of resolving different hydrogen-related vibrations. The peak intensities increase as the electron probe is moved closer to the specimen edge, as shown in Fig. 2b. Supplementary Fig. 1 is a dark-field image showing the positions of the electron probe. It is interesting to note that vibrational signals can still be detected with a probe 100-nm away from the specimen. Figure 2d presents details of the intensity variations of the C=O intensity, along a line scan shown in the annular dark field image Fig. 2c, with points every 0.5 nm. Also shown in Fig. 2d is the integrated intensity encompassing the C–H, N–H and NH_2_ stretches.

Energy and momentum conservation leads to a scattering wavevector along the beam direction, *q*_*z*_, that depends on the energy loss, Δ*E* such that:


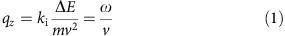


where *v* is the electron velocity, *m* is the electron mass, *k*_i_ is the initial wavevector for the fast electron and *ω*=Δ*E/ħ* is the frequency of vibration.

Together with the condition in equation (1), the classical dielectric response theory for an electron beam running parallel to a slab a distance *d* from the edge gives a functional dependence of


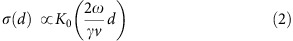


for the scattering cross-section *σ*(*d*), where *K*_0_ is a Bessel function of the second kind^25^,^26^ and *γ* is the relativistic Lorentz factor.

Beyond 30 nm from the edge the intensity variation is very well described by equation (2), based on classical dielectric theory. The deviation from the theoretical model near the sample, especially in the integrated counts of the peak due to vibrations associated with hydrogen, is due to beam damage.

### The ultraviolet energy range

**Figure 3:**
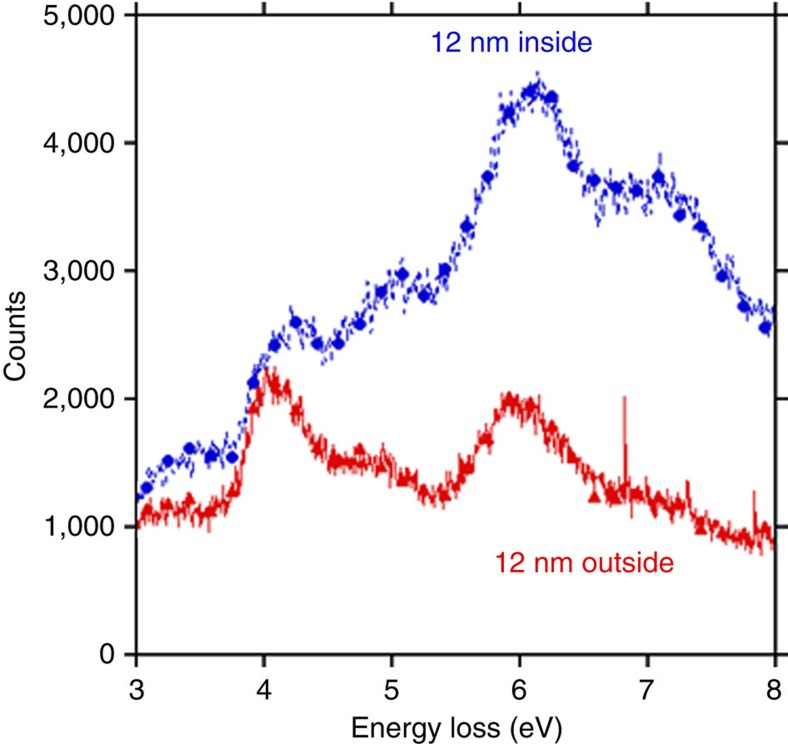
Ultraviolet region spectra with the beam inside and outside the sample. Comparison of EEL spectra of the ultraviolet region, taken with probe 12 nm inside and 12 nm outside the sample in the vacuum region. The spectrum with the probe outside the specimen lacks the high-energy features at ∼7 eV that are believed to be responsible for ionization. The ratio of the peaks at 4 and 6 eV changes when moving from outside to inside the specimen, since lower-energy peaks can be excited at greater distance from the specimen edge.

In contrast to most optical probes, EELS covers a broad spectral range, and spectral characteristics of both the infrared and ultraviolet–visible regions can in principle be recorded simultaneously. Figure 3 focuses on the ultraviolet range, comparing spectra with the probe at positions 12 nm inside the sample and 12-nm away from the edge. Strong peaks at 4.04 and 6 eV are observed, with a weaker peak at 4.94 eV, superimposed on a broad spectral band. These features have been observed in the dielectric response derived from ultraviolet ellipsometry of thin films of guanine on Si(111) substrates^27^, and polarized reflection spectra of 9-ethylguanine and guanine hydrochlodirde dihydrate^28^. The 4.04 eV peak is due to π–π* transitions from the highest occupied molecular orbital to the lowest unoccupied molecular orbital as supported by both self consistent field^29^ and density functional theory^30^ calculations. The 6 eV peak is due to transitions from the highest occupied molecular orbital to a σ* level. The broad band is associated with the tails of a π-collective plasmonic excitation. The 4–6 eV peaks become visible when the probe is within 15 nm of the edge, but as shown in Supplementary Fig. 2 the intensity of higher-energy loss features increases significantly only when the electron probe is very close to the specimen edge.

### Damage-free vibrational spectroscopy

**Figure 4:**
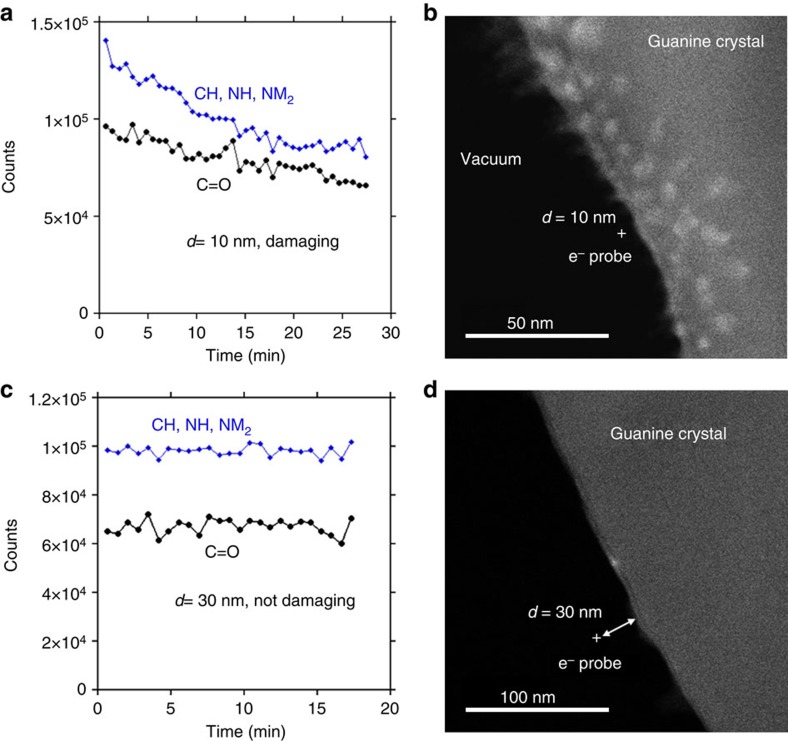
Time dependence of infrared region peaks. The time dependence of the C=O peak height and the integrated peak area from the hydrogen-stretching modes for an electron probe placed (**a**) 10 nm from the edge of the specimen and (**c**) 30 nm from the edge of the specimen. Images taken after acquiring the data in **a**,**c** are shown in **b**,**d**, respectively. Note the visible signs of damage when the probe was positioned 10 nm from the edge of the specimen, in contrast to the absence of damage when the probe was at a distance of 30 nm. Also note that damage is present at distances greater than would be expected from the ‘delocalization' of the probe 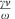.

Figure 4a,c shows how the intensity of vibrational EELS signals varies with time for a beam situated at a fixed position. A slow decay is observed for *d*=10 nm, taking place on a timescale of 30 min. The time for the intensity to decay by a factor of *1*/*e* is 53 min for the bonds of carbon and nitrogen with hydrogen and 68 min for the carbon oxygen bond. This would imply that hydrogen is broken off in the first stage of radiation damage. Visible signs of damage are also observed in the image taken immediately after this data was acquired (Fig. 4b). These patches are similar in appearance to what is observed when the electron probe is stationary on the specimen for 20 s as shown in Supplementary Fig. 3. Even with a low electron flux of 0.02 electrons per nm^2^ per s, as would be typical in cryo-electron microscopy, there is little high-resolution structural information remaining after exposure for just over 1 min, as shown in Supplementary Fig. 4.

It would be expected that the lateral distribution of damage would be confined to ∼10 nm, which is the distance of the probe from the sample edge and hence the scale of dominant interaction. However, damage is also observed further away in this case. In a discussion on damage caused by fast secondary electrons^31^,^32^,^33^, it has been proposed that it mainly originates from K shell excitations. For a beam 10 nm outside the specimen, it is not possible to excite K edges; hence the electrons causing this damage very likely originate from lower-energy excitations. Remarkably, and in contrast to the measurement at 10 nm distance, no damage is observed when the beam is 30 nm from the edge, see Fig. 4 c,d. The reason for this striking difference between the two beam positions is that a critical energy is crossed at this *d*-range: the ionization energy of guanine is ∼7 eV, which translates into ∼14 nm (refs 30, 34).

## Discussion

The lateral spatial resolution is approximately given by the distance of the electron beam from the specimen edge. In the electron microscopy of beam-sensitive specimens, spatial resolution is limited by the need to restrict the electron beam fluence to avoid damage^9^. In this sense, aloof beam spectroscopy is similar to other forms of electron microscopy and spectroscopy in that there is a tradeoff between damage and spatial resolution. An advantage of aloof beam spectroscopy is that vibrational spectral features can be safely detected before bringing the beam closer to the sample and before damage becomes apparent. Furthermore, it is possible to directly monitor which bonds are being broken given that radiation damage often involves ejection of hydrogen, either by a knockon impact process or by radiolysis. By resolving the infrared signatures of hydrogen attached to different atoms as the electron probe gets closer to the specimen within a range of 10 nm, the sensitivity of radiation damage to ionization events of different energies can be explored.

In practical applications aloof beam spectroscopy will be complementary to tip-enhanced infrared absorption. The main difference is the nature of the specimen. Flat films are better studied using scanning probe infrared absorption, while aloof beam EELS is more appropriate for TEM specimens where the features of interest lie close to an edge. The spatial resolution of electron beam techniques applied to biological specimens is limited by the ability to tolerate damage. If high resolution is required, the electron beam can be moved very close to, or even on to, the specimen for brief periods to achieve nanometre or better resolution. The spatial resolution of tip-enhanced infrared absorption is approximately the tip radius of ∼20 nm, as demonstrated by Huth *et al*.^5^. At present, tip-enhanced infrared spectroscopy has superior energy resolution, but the spectral range is limited by the laser power to 600 cm^−1^ (75 meV). In electron spectroscopy (within the electron microscope), excitations of all energies take place simultaneously, from vibrational modes to plasmons and single-electron excitations with energies equivalent to ultraviolet and soft X-ray photons. We have shown that by controlling the distance of an external narrow electron probe from the edge of a specimen, we can selectively probe vibrational modes without exciting energies above the energy thresholds that potentially lead to radiation damage. This can be seen graphically in Supplementary Fig. 5, which shows strong infrared peaks when the electron probe is outside the specimen while the features corresponding to ultraviolet excitations are hardly detectable. Moreover, by stepping outside the sample, we avoid high-momentum transfer impact processes (knock on damage) that eject or displace light atoms. This is especially significant for biological samples that are predominately composed of light elements. Notably, in contrast to optical spectroscopy, we still retain all the advantages of electron microscopy, with fast and easy control of the electron beam, and with the ability to easily change *d*-values or move to a different area on the sample.

The superior high-energy resolution provided by the present monochromated electron microscope^35^ is an important ingredient of the proposed approach. For example, we have successfully distinguished the vibrational modes of hydrogen covalently bonded to carbon versus nitrogen and clearly resolved the peak due to stretching of the C=O bond. Further improvements in the energy resolution will make it possible to record all peaks corresponding to modes with frequencies greater than ∼50 meV (400 cm^−1^), encompassing much of the useful range in infrared absorption spectroscopy.

To realize its full potential for characterizing biological specimens damage free, aloof beam EELS should be combined with cryo-TEM. Complementary information can then be obtained from low-dose imaging and aloof beam electron spectroscopy. We have already resolved the C=O stretch, one of the amide peaks characteristic of proteins. An improvement in resolution to 5 meV would make it possible to clearly resolve the other amide peaks^36^ and analyse the secondary structure of proteins in the electron microscope in a damage-free mode, while determining overall morphology using standard low-dose imaging techniques in the same instrument. Using the NH stretch vibrational mode, it should be possible to localize proteins in membranes at nanometre resolution. Exciting possibilities of distinguishing other significant biological molecules from their vibrational or optical signature are expected to be realized by our approach, enabling nanometre-level resolution of functional groups in biological structures.

## Methods

### Specimen preparation

Japanese Koi fish (*Cyprinus capio*) were purchased from Peka (Rehovot, Israel) and were maintained in an aquarium at the animal care facility in the Weizmann Institute of Science.

### Guanine crystals extraction

Fish scales were mechanically removed and washed with double deionized water (DDW). The guanine crystals were extracted by detaching the skin from underneath the scale in a small volume of DDW, dispersing the crystals into the water. The crystal suspension was collected and centrifuged (10 min, 10,600 r.p.m., Eppendorf centrifuge 5417C). The supernatant was replaced with DDW, the crystal pellet was resuspended and the procedure was repeated twice more.

### TEM grids

The crystal suspension was dispersed on a Cu holy carbon-coated TEM grid (MultiA, Quantifoil), and was left in vacuum until completely dehydrated.

### FTIR

Synthetic anhydrous guanine crystals (Sigma Aldrich) was transferred to an agate mortar and was lightly crushed with KBr and a pellet was prepared. The spectra were recorded on a NICOLET is5 Fourier transform infrared (FTIR) spectrometer. The FTIR shows a splitting of the C=O peak, since the C=O are in two different environments.

### EELS

Spectra, with the exception of the spectrum shown in Fig. 2a, were acquired at Arizona State University with a Nion High-Resolution Monochromated EELS STEM (HERMES) system consisting of a Nion STEM100 scanning transmission electron microscope (STEM) equipped with a Nion high-energy resolution monochromator and a modified Gatan Enfinium EEL spectrometer. The energy resolution, measured from the half width of the zero loss peak (ZLP), was 15 meV. The accelerating voltage was 60 kV, the probe current ∼1 pA, the beam convergence was 12 mrad and the electron probe diameter was ∼2 nm. A 1-mm spectrometer entrance aperture was used, defining a collection angle of 15 mrad.

As a first stage, the raw spectra were corrected for energy drift, which was a major problem in all acquisitions with high dispersion. Since the ZLP was saturated, the centroid was found by assessing where the intensity was 5% lower than its peak value and taking the mean of these two energies. This procedure corrected drift to within one or two channels. Acquisition parameters for the spectrum in Fig. 2b were 40 s and 2 meV per channel, and for the spectrum shown in Fig. 3 were 7 s and 5 meV per channel.

The spectrum shown in Fig. 2a was acquired using the Nion HERMES system at Rutgers University. It was equipped with a Nion prototype spectrometer with first- to fourth-order aberration correction and a 2k × 2k pixel lens optically coupled with CMOS camera. The scintillator is constructed such that the ZLP completely missed the scintillator, and hence the ZLP tail due to light spread in the scintillator was greatly suppressed. Supplementary Fig. 6 shows how this significantly reduces the background intensity. A power-law background was fitted between 66.7 and 96.13 meV. The EELS dispersion was 0.46 meV per channel, and the energy resolution (half width of the ZLP, measured in a spectrum recorded separately) was 8 meV. The accelerating voltage was 60 kV, the probe diameter was ∼2 nm and the probe current ∼2 pA. The probe convergence semi-angle was 15 mrad, and the EELS collection semi-angle was 13 mrad. The spectrum shown in Fig. 2a is an average of 19 energy-aligned spectra, each with 10 s acquisition time, taken from a point 30 nm from the edge of the guanine crystal.

### TEM diffraction

TEM diffraction of the guanine crystals was conducted on an FEI Tecnai F20 microscope with the beam spread out over micron-sized regions at room temperature. Electron diffraction patterns were recorded on a Gatan US4000 charge-coupled device camera.

### Spectrum processing

With the exception of the spectrum shown in Fig. 2a, separate backgrounds were fit under the C=O peak, and the CH, NH and NH_2_ peaks. The fitting regions were 0.16–0.18 and 0.23–0.25 eV for the C=O peak, and 0.25–0.29 and 0.45 eV–0.52 eV for the stretching modes involving hydrogen. The C=O peak was the intensity remaining from 0.18 to 0.23 eV after background subtraction, and the peak corresponding the CH, NH and NH_2_ stretches was the intensity remaining from 0.29 to 0.45 eV after background subtraction. These energy windows were used for the integrated intensities plotted in Figs 2d and 4a,b, though the window for fitting the power-law background under the compound CH, NH and NH_2_ stretch peaks was modified to stop at 0.48 eV and the integration window ended at 0.43 eV, due to problems with a defective pixels in the detector array in the spectra used for Fig. 2d. Supplementary Fig. 7 shows the raw and background subtracted spectra.

## Additional information

**How to cite this article:** Rez, P. *et al*. Damage-free vibrational spectroscopy of biological materials in the electron microscope. *Nat. Commun.* 7:10945 doi: 10.1038/ncomms10945 (2016).

## Supplementary Material

Supplementary Information Supplementary Figures 1-7 and Supplementary References.

## Figures and Tables

**Table 1:** Assignment of peaks observed by aloof EELS in the infrared region.

**Peak**	**Energy (meV)**	**Frequency (cm**^**−1**^)	**Assignment**
a	209	1,666	C=O stretch
b	334	2,663	C–H stretch
c	357	2,846	N–H stretch
d	386	3,078	Symmetric NH_2_
e	411	3,277	Antisymmetric NH_2_

EELS, electron energy loss spectroscopy.
