# Erratum: Damage-free vibrational spectroscopy of biological materials in the electron microscope

**DOI:** 10.1038/ncomms11592

**Published:** 2016-05-04

**Authors:** Peter Rez, Toshihiro Aoki, Katia March, Dvir Gur, Ondrej L. Krivanek, Niklas Dellby, Tracy C. Lovejoy, Sharon G. Wolf, Hagai Cohen

Nature Communications 7, Article number: 10945 10.1038/ncomms10945 (2016); Published: 03102016; Updated: 05042016

The author Hagai Cohen is incorrectly omitted from the list of corresponding authors. The corresponding authors are Peter Rez and Hagai Cohen. The correct information for correspondence is: ‘Correspondence and requests for materials should be addressed to P.R. (Peter.Rez@asu.edu) or to H.C. (Hagai.Cohen@Weizmann.ac.il).'

