# Supplementary material for: Damage-free vibrational spectroscopy of biological materials in the electron microscope
Source: Nat Commun. 2016 Mar 10;7:10945. doi: 10.1038/ncomms10945 (PMC4792949; doi:10.1038/ncomms10945)
Supplement: Supplementary Information — Supplementary Figures 1-7 and Supplementary References. [file ncomms10945-s1.pdf]

Supplementary Information

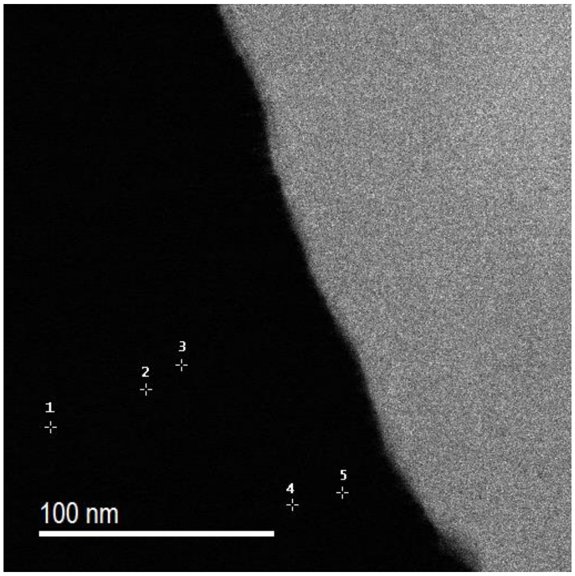

Supplementary Figure 1. **Electron Probe Positions**

Dark Field image showing beam positions for Fig 2b.

Supplementary Figure 1 shows the positions used for acquiring the spectra shown in Figure 2b. The beam was moved to sample a fresh area after point 3 at 30nm.

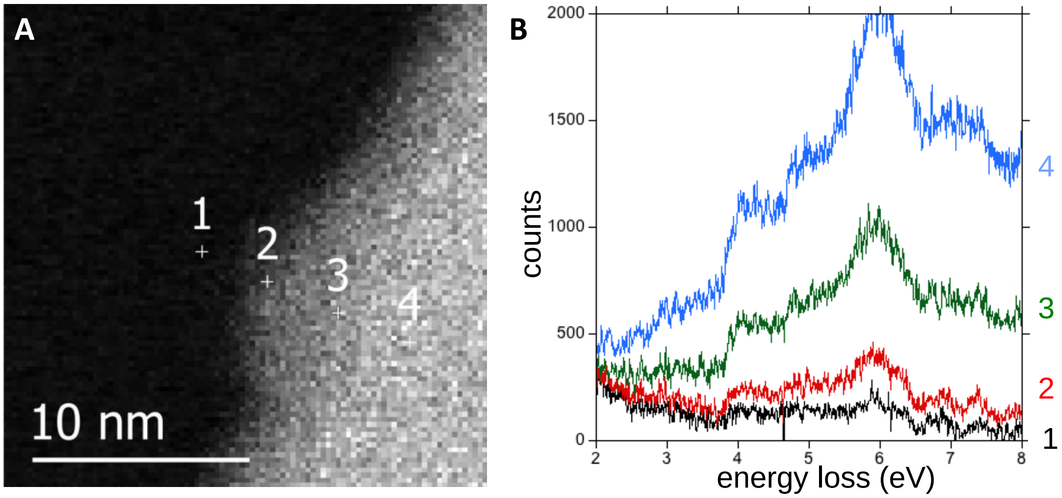

Supplementary Figure 2 Variation of UV Region Spectra with Electron Probe Position

EEL Spectra in the visible and UV region as the beam is moved closer to the specimen.

Supplementary Figure 2 shows the EELS signal in the visible and UV region as the beam is moved closer and into the sample. The positions for the spectra are marked in the image. Note that the peak at 7 eV, only appears when the beam is inside the specimen. The ratio of the heights of the 4.04 eV and 6 eV peaks changes as the beam moves into the specimen and the background from the π plasmon becomes more apparent.

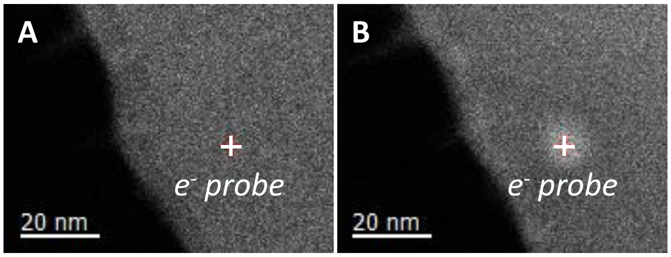

Supplementary Figure 3. Damage from stationary electron probe

(**a**) Dark field image of the guanine crystal after one quick scan and (**b**) after leaving the probe on the crystal for 20 secs—beam damage is apparent.

Supplementary Figure 3 shows a dark field image of the guanine crystal after one quick scan (**a**) and after leaving the probe on the crystal for 20 secs (**b**)—beam damage is apparent in (**b)**

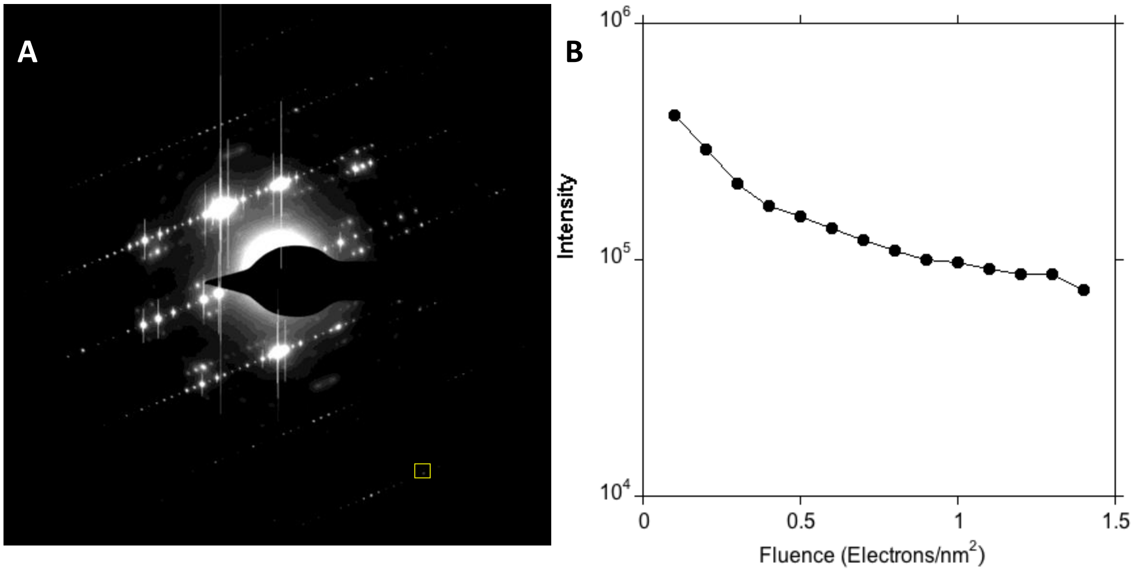

Supplementary Figure 4. Decay of high order diffraction spot intensity

(a) electron diffraction pattern (**b**) decay of (0,16,7) spot highlighted in (**a)** with exposure to electrons.

The traditional way of measuring radiation sensitivity is to plot the intensity of a diffraction spot as a function of accumulated dose (strictly fluence). The diffraction pattern from the fish scales is shown as Supplementary Figure 4a. The pattern was indexed according to the cell for β guanine proposed by Hirsch et al [1]. The (010) direction is the same as in the cell published by Guille and Clegg [2]. Supplementary Figure 4b shows a logarithmic plot of the intensity of the (0,16,7) spot (shown in yellow box) as a function of accumulated fluence at an electron exposure rate of 0.02 electrons/nm^2/sec. The fluence needed to reduce the intensity by 1/e is 0.5 e /nm^2 which is equivalent to a dose of 8 x10^{-6} C/cm^2. This is much lower than the fluence quoted by Siangchaew and Libera for polystyrene which was measured from changes in the π peak intensity [3,4] It is also lower than the equivalent fluence published by Li and Egerton[5] for various aromatic compounds measured from both changes in the π peak intensity and reduction in the intensity of the strongest diffraction spot. This is no doubt due to the higher order reflection being more sensitive to high resolution structural information that is strongly affected by displacements of the guanine molecules within the unit cell.

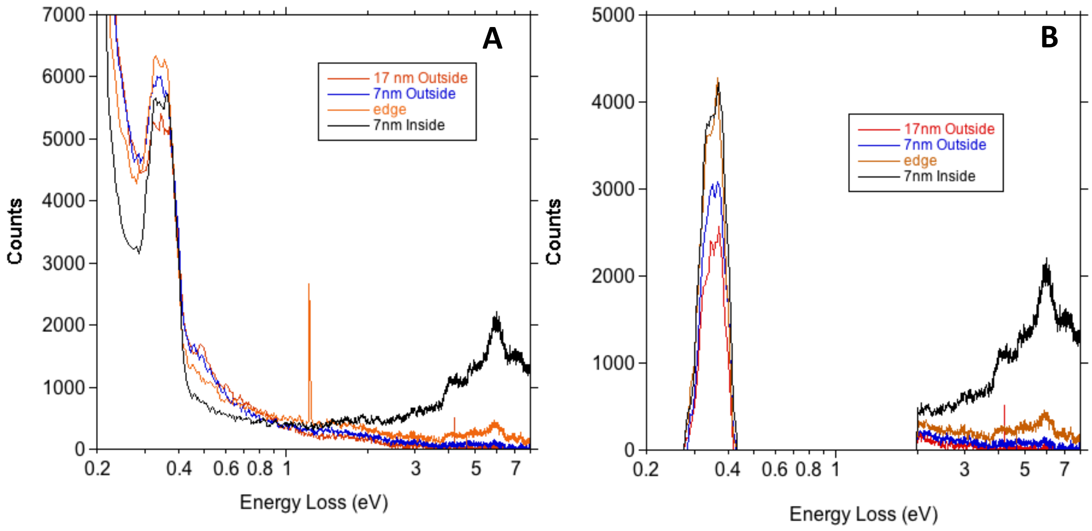

Supplementary Figure 5. IR and UV regions on the same scale.

Supplementary Figure 5a shows the raw spectra, Supplementary Figure 5b shows the spectra after background subtraction using the procedures described in the methods section.

Supplementary Figure 5 shows the IR peak due to CH, NH and NH_2 stretches, and the UV peaks on the same scale. The dispersion was the same as that used for the UV region and a logarithmic energy scale was needed to accommodate the energy range. The raw spectra are shown as Supplementary Figure 5a, the relative magnitudes are more apparent from the background subtracted spectra shown as Supplementary Figure 5b. The intensity of the IR peak has halved as the probe is moved to a position 17nm outside the specimen, while the intensity of the damage causing UV peaks has almost disappeared.

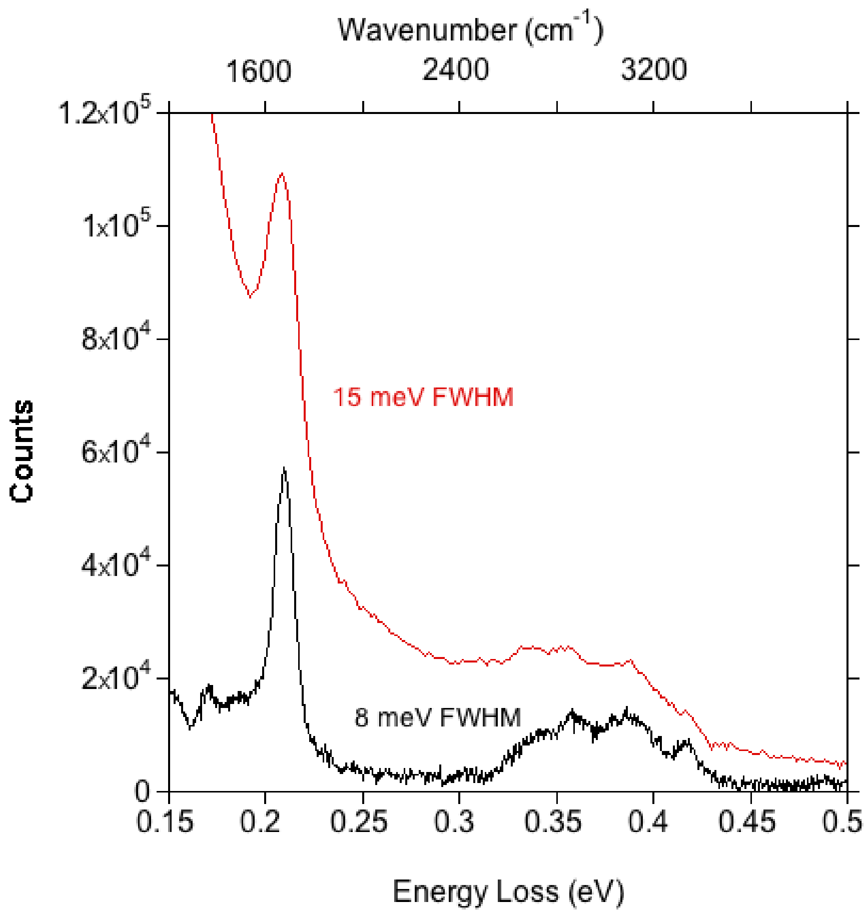

Supplementary Figure 6. **IR Spectra from ASU and Rutgers Instrument**

Comparison of IR region spectrum taken using the ASU instrument (red, 15 meV FWHM zero loss) and the Rutgers instrument (black, 8 meV FWHM zero loss).

Supplementary Figure 6 shows a comparison between the spectrum acquired at ASU (15 meV FWHM) and the higher energy resolution spectrum acquired at Rutgers (8 meV FWHM). This graphically illustrates that the tail of the zero loss peak is the background in the IR region. Special efforts were taken in the design of the Rutgers spectrometer to improve the zero loss FWHM, and to reduce the tail of zero loss peak. These almost eliminate the background under the CH, NH and NH_2 stretch peaks in the unsubtracted/raw spectrum.

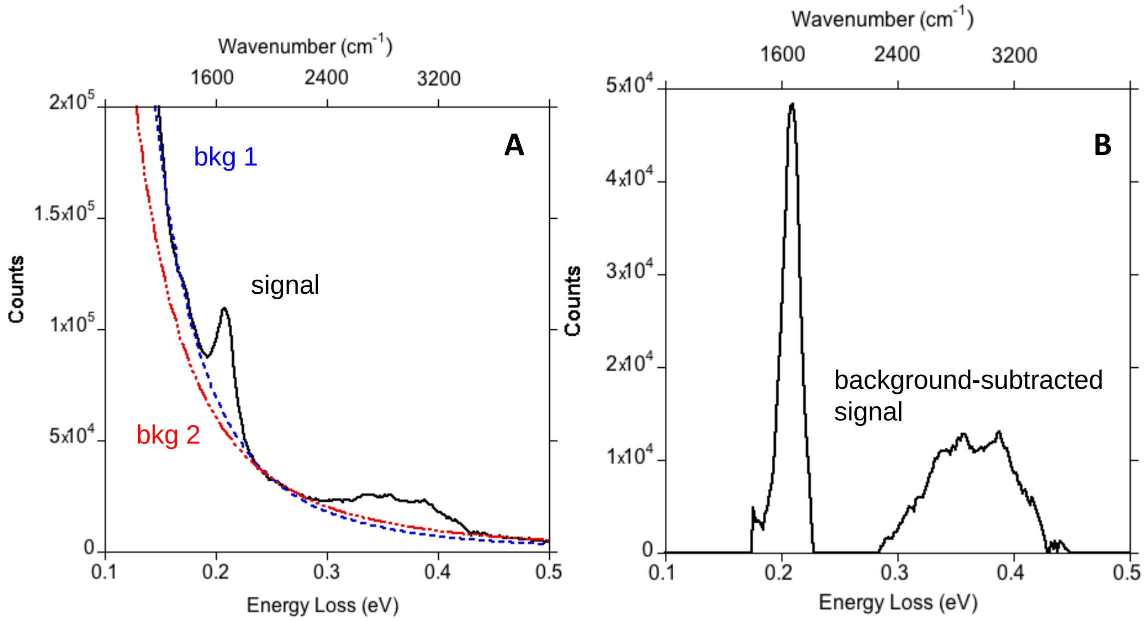

Supplementary Figure 7   Background Fitting Procedure

(**a**) Energy drift corrected spectrum showing the signal from the IR peaks on top of a background from the zero loss peak, and two separate background models used for the first peak and second peak, respectively (**b**) background-subtracted spectra.

Supplementary Figure 7 shows the background fitting procedure used to extract the peak intensities shown in Figs 2b, 2d, 4a and 4c. Separate power law fits (see Methods section) were used for the C=O peak and the composite peak from CH, NH and NH_2 vibrations.

Supplementary References

1 Hirsch, A. *et al.* 'Guanigma': the revised structure of biogenic anhydrous guanine. *Chem. Mat.*, doi:10.102/acs.chemmater.5b03549 (2015).

2 Guille, K. & Clegg, W. Anhydrous guanine: a synchrotron study. *Acta Cryst. C* **62**, 515-517 (2006).

3 Siangchaew, K. & Libera, M. The influence of fast secondary electrons on the aromatic structure of polystyrene. *Phil. Mag. A* **80**, 1001-1016 (2000).

4 Egerton, R. F., Lazar, S. & Libera, M. Delocalized radiation damage in polymers. *Micron* **43**, 2-7 (2012).

5 Li, P. & Egerton, R. F. Radiation Damage in Coronene, Rubrene and p-Terphenyl, measured for incident electrons of kinetic energy between 100 and 200 keV. *Ultramicroscopy* **101**, 161-172 (2004).
